# Impact of End-of-Life Circumstances on the Adjustment of Bereaved Siblings of Children Who Died from Cancer

**DOI:** 10.1007/s10880-021-09797-x

**Published:** 2021-06-26

**Authors:** Ansley E. Kenney, Perri R. Tutelman, Rachel S. Fisher, Keagan G. Lipak, Maru Barrera, Mary Jo Gilmer, Diane Fairclough, Terrah Foster Akard, Bruce E. Compas, Betty Davies, Nancy S. Hogan, Kathryn Vannatta, Cynthia A. Gerhardt

**Affiliations:** 1 Center for Biobehavioral Health, The Abigail Wexner Research Institute at Nationwide Children’s Hospital, Columbus, OH, USA; 2 Dalhousie University, Halifax, NS, Canada; 3 The Hospital for Sick Children, Toronto, ON, Canada; 4 University of Toronto, Toronto, ON, Canada; 5 Vanderbilt University School of Nursing, Nashville, TN, USA; 6 Colorado School of Public Health, Aurora, CO, USA; 7 Department of Psychology and Human Development, Vanderbilt University, Nashville, TN, USA; 8 University of Victoria, Victoria, BC, Canada; 9 Professor Emerita, Loyola University Chicago, Chicago, IL, USA; 10 Department of Pediatrics, The Ohio State University, Columbus, OH, USA

**Keywords:** Sibling Bereavement, Cancer, End of Life, Adjustment

## Abstract

**Objective:**

To examine the impact of end-of-life (EoL) circumstances on grief and internalizing symptoms among bereaved siblings.

**Methods:**

Bereaved families (*N* = 88) were recruited from three sites 3–12 months (*M*=11.57, *SD*=3.48) after their child’s death from cancer. One sibling per family aged 8–17 years (*M*=12.41, *SD*=2.64) was randomly selected to participate. Families completed measures of siblings’ grief and internalizing symptoms, as well as a structured interview about circumstances surrounding the death.

**Results:**

Mother and sibling reports of EoL circumstances were generally concordant, except there was a discrepancy between mothers and children about whether or not children expected their sibling’s death (*t*(75) = 1.52, *p*=0.018). Mother-reports of sibling internalizing symptoms were above the normative mean (*t*(83) = 4.44, *p*≤0.001 (*M* = 56.01 ± 12.48), with 39% (*n*=33) in the borderline/clinical range. Sibling opportunity to say goodbye was associated with greater grief-related growth (*t*(79) = −1.95, *p* = 0.05). Presence at the death and wishing they had done something differently were both associated with greater grief (*t*(80) = −2.08, *p*=0.04 and *t*(80) = −2.24, *p*=0.028, respectively) and grief-related growth (*t*(80) = −2.01, *p*=0.048 and *t*(80) = −2.31, *p*=0.024, respectively). However, findings were primarily unique to sibling-report, with few mother-reported effects.

**Conclusions:**

The adjustment of bereaved siblings may be affected by certain modifiable circumstances surrounding the death of their brother or sister. A proportion of bereaved siblings had elevated internalizing symptoms irrespective of circumstances at EoL. Further work is needed to understand predictors of adjustment among bereaved siblings to provide better support and optimize their outcomes.

Cancer is the leading disease-related cause of death for children and adolescents (Siegel, Miller, & Jemal, 2019). The death of a child is devastating and often associated with profound grief by bereaved family members. Approximately 80% of families have more than one child with siblings sharing a unique, powerful, and often lifelong bond (Kreider, 2007). Siblings endure significant stress when a child is diagnosed with cancer and are at risk for poor psychosocial outcomes when their brother or sister dies (Gerhardt, Lehmann, Long, & Alderfer, 2015; Hoffmann, Kaiser, & Kersting, 2018).

Studies suggest that siblings bereaved from childhood cancer may have elevated externalizing and internalizing problems compared to normative values as reported by parents and teachers (Birenbaum, 2000; Howard Sharp et al., 2018). Although severe emotional and behavioral problems are uncommon (Hoffmann et al., 2018), bereaved siblings report sadness, loneliness, anxiety, and guilt (Foster et al., 2012; Nolbris & Hellstrom, 2005; Rosenberg et al., 2015), as well as difficulties with peer and family relationships (Foster et al., 2012; Gerhardt et al., 2015). Studies have also documented the grief experienced by bereaved siblings (Foster et al., 2012; Howard Sharp et al., 2018; Nolbris, Enskär, & Hellstrom, 2014; Thompson et al., 2011), which can persist or recur many years later (Lövgren et al., 2018; Sveen, Eilegård Wallin, Steineck, & Kreicbergs, 2014). However, not all bereaved siblings experience prolonged difficulties, as some report personal (e.g., maturity, new life perspective, academic goals) and family (e.g., improved communication, strengthened bonds) growth following their brother’s or sister’s death (Akard et al., 2019; Foster et al., 2012; Rosenberg et al., 2015). Indeed, grief and grief-related growth can co-occur (Hogan & De Santis, 1996; Hogan, Schmidt, & Coolican, 2014; Hogan & Greenfied, 1991). Grief-related growth comprises positive change or personal growth resulting from the grief experience (Hogan & Schmidt, 2002).

Research on predictors of psychological adjustment among bereaved siblings is scarce. A systematic review of outcomes among bereaved parents after a child’s death from cancer found that factors such as parental perceptions of the quality of their child’s medical care and death, parent preparedness for the death, and location where the child died were associated with psychosocial morbidity (Rosenberg, Baker, Syrjala, & Wolfe, 2012). While it is unclear if these factors are relevant for sibling outcomes, emerging work suggests that the circumstances surrounding the child’s death may play an important role in optimizing sibling psychological outcomes given that most are modifiable in nature. In retrospective samples of adolescent and young adult siblings bereaved up to 17 years earlier, psychological stress and grief were predicted by end-of-life (EoL) variables such as the perception that their brother or sister did not have a good death, limited information and preparation for the death, and the missed opportunity to say goodbye (Lövgren et al., 2018; Rosenberg et al., 2015).

Cancer-bereaved siblings have reported desires to be informed when their brother or sister is nearing EoL and to be involved in their care (Nolbris & Hellstrom, 2005; Steele et al., 2013). Indeed, past guidelines have recommended that siblings be included during the terminal phase of the illness and provided the opportunity to be involved in decisions related to the child (Spinetta et al., 1999). However, literature on the role of EoL circumstances on sibling adjustment is limited by its retrospective and qualitative focus on adolescent and young adult siblings, who have been bereaved for several years to decades. Children’s understanding of death follows a developmental progression which may influence how they understand the death of their sibling and thus their grief reaction (Kenyon, 2001). Understanding of the core concepts of death (e.g., inevitability, causality, irreversibility) are not assumed until around age ten, which may be variable and may not be developed until later on in some children and adolescents (Kenyon, 2001). Furthermore, there are differences in the dimensions of grief between children and young adults, which may reflect differences in life experiences and cognitive ability (Melhem, Moritz, Walker, Shear, & Brent, 2007). Research capturing the impact of EoL circumstances on school-aged and adolescent siblings soon after the child’s death is needed. Thus, we examined the associations between EoL circumstances and internalizing symptoms, grief, and growth, among bereaved siblings of children who died from cancer. We hypothesized that EoL circumstances (e.g., anticipation of the death, saying goodbye, and home-based deaths) would be associated with less sibling grief and internalizing symptoms and more grief-related growth.

## Methods

This research was part of a multi-site longitudinal study of families following the death of a child from cancer (Gerhardt et al., 2012). In the larger study, data collection involved visits to bereaved siblings’ schools and homes during the first year after the death, followed by another home visit one year later. Data collection occurred from 2006 to 2012. This paper includes cross-sectional data collected from families at the first home visit.

### Procedures

Institutional Review Board approval was obtained at three participating children’s hospitals in the United States and Canada. Bereaved families were identified from cancer registries at each site. Families were mailed a letter from the deceased child’s attending oncologist introducing the study 3 to 12 months after the child’s death. Research staff then phoned families to describe the study, confirm eligibility, and randomly select one sibling from each family for participation. Home visits for interested families were scheduled as soon as they could be arranged after the initial school visit. Informed consent and assent were obtained from parents and siblings, respectively, at the home visit. Trained research assistants administered questionnaires and structured interviews related to the circumstances surrounding the child’s death to bereaved parents and siblings, separately.

### Participants

At recruitment, families were eligible if they: (a) had a bereaved sibling between 8 to 17 years old and in school without special education, (c) were English-speaking, and (d) lived within 100-miles of the hospital. Adopted, half-siblings, and step-siblings (referred to as siblings) were eligible as long as the parent reported the sibling had regular, ongoing contact with the deceased child. Parents of 169 bereaved siblings meeting eligibility requirements were initially contacted for recruitment. Of these, 47 (28%) declined, and 122 (72%) permitted school contact. Separate data were collected in the schools of 105 (86%) bereaved siblings. Of these, 10 families were not followed due to the relocation of the principal investigator. Of the remaining 95 families, 89% (*n* = 85) participated in the home visit. An additional 3 siblings for whom school data were not collected also participated in a home visit, resulting in a final sample of 88 bereaved families (52%). All 88 families completed the initial grief interview and measures for the study. Only mother and sibling data are reported in this paper due to fewer fathers participating. Siblings were on average 12.41 years of age (*SD* = 2.64, Range 8–18 years), and 73% (*n* = 62) identified as White. Mothers were on average 40 years of age (*SD* = 6.57, Range 27–60 years). Time since the death of the child at the family home visit averaged 11.57 months (*SD* = 3.48, Range 6–24 months). See Table 1 for background characteristics.

### Measures

#### End of Life Circumstances.

Mothers (*N* = 88) and siblings (*N* = 88) independently completed a structured initial grief interview developed for this study by the principal investigators detailing the circumstances surrounding their child’s death. Questions included parent/sibling anticipation of the child’s death, who informed the sibling that the child had died, place of death, parent/sibling presence during the death, whether the parent/sibling had an opportunity to say goodbye to the child before death, and if the sibling wished they would have done anything differently around the time of the death. Following participant responses to each interview question, interviewers selected a code. If initial responses did not yield enough information to code the response, follow-up probes were asked (e.g., did you have an opportunity to say goodbye? Probe: was this in person or did it happen some other way?). A summary of the questions and coding of responses can be found in the Appendix.

#### Internalizing Symptoms.

Mothers described the bereaved siblings’ internalizing symptoms using the Child Behavioral Checklist (CBCL) (Achenbach, 1991). The CBCL is a 118-item parent-report measure of emotional and behavioral problems for children 6 to 18 years of age. Items are scored on a 3-point scale based on their frequency in the last 6 months ranging from 0 (not true) to 2 (very true/often true). *T* scores are calculated based on a nationally representative sample of children and youth. The Internalizing Symptoms *T* score (comprising the Anxious/Depressed, Withdrawn/Depressed, and Somatic Complaints scales) was used for this paper. Reliability and validity are well established (Achenbach, 1991).

#### Grief and Personal Growth.

Bereaved siblings completed the Hogan Sibling Inventory of Bereavement (HSIB), a 46-item questionnaire assessing siblings’ responses to the death of a brother or sister (Hogan & De Santis, 1996; Hogan et al., 2019). The measure is comprised of 2 subscales: Grief (e.g., “I have no control over my sadness”) and Personal Growth (e.g., “I try to be kinder to other people”). Items are scored on a 5-point scale ranging from 1 (does not describe me at all) to 5 (describes me very well) based on the past 2 weeks. Items on each subscale are summed. Both subscales have strong reliability and validity (Hogan et al., 2019).

#### Medical Chart Review.

Information regarding the deceased child (e.g., diagnosis, date of death) was extracted from medical records by trained research staff.

### Analyses

Descriptive statistics characterized the sample as well as mother and sibling reported factors surrounding the death, including anticipation of the death, informant of child’s death to sibling, location of death, sibling presence at time of child’s death, and parent and sibling opportunity to say goodbye. Chi-square tests examined concordance between mother and sibling responses to the initial grief interview and *t*-tests for those found to be significantly discrepant. Pearson correlations examined associations between sibling age, months since the child’s death, and the primary outcome variables of internalizing symptoms and sibling-reported grief and grief-related growth. Categorical items on the initial grief interview were collapsed into dichotomous variables (i.e., present/nearby vs. not present, expected/expected, but not at that time vs. unexpected, etc.) due to low frequency responses in some categories. One sample *t*-test compared sibling internalizing symptoms against the normative mean (*M* = 50, *SD* = 10). Independent *t*-tests (i.e., dichotomized variables, location of death, etc.) and analysis of variance (ANOVA) (i.e., opportunity to say goodbye, etc.) examined differences in mother-reported internalizing symptoms and sibling-reported grief and growth across both mother and sibling reported circumstances surrounding EoL.

## Results

### Descriptive Analyses

#### Circumstances Surrounding the Death Reported by Mothers.

Bereaved caregivers were asked questions regarding circumstances surrounding the death, including mother-reported sibling experiences. Ninety percent of mothers (*n* = 75) and 78% (*n* = 65) of siblings as reported by mothers were aware in advance that the child was dying, (median = 30.0 and 30.0 days, respectively). According to mother report, most siblings were informed of the child’s death by parents (70%; *n* = 49), 10% (*n* = 7) medical staff, and 20% (*n* = 14) others or unsure. Half of deaths took place at home (52%, *n* = 43), with the remaining in the hospital (43%, *n* = 36), or other location (5%, *n* = 4). Thirty-five percent (*n* = 29) of siblings were present at the death, 33% (*n* = 27) nearby, or 35% (*n* = 27) not present. Mothers reported that 60% (*n* = 49) of siblings and 85% (*n* = 70) of mothers had the opportunity to say goodbye. Circumstances of sibling anticipation of the death (age, *p* = 0.17 and sex, *p* = 0.74), presence at death (age, *p* = 0.57 and sex, *p* = 0.34), or opportunity to say goodbye (age, *p* = 0.11 and sex, *p* = 0.69) did not vary by sibling age or sex. See Table 2 for mother-reported and sibling-reported circumstances surrounding EoL.

#### Circumstances Surrounding the Death Reported by Siblings.

Bereaved siblings were also asked about the circumstances surrounding their brother’s or sister’s death. Fifty-eight percent (*n* = 46) of siblings reported they knew that their brother/sister was going to die (*M* = 44.79 ± 62.99 days). Sixty percent of siblings (*n* = 28) said their parents told them, 2% (*n* = 1) said medical staff, and 38% (*n* = 18) said other (e.g., other family member or friend) informed them of the death. Approximately 36% (*n* = 30) of siblings reported they were present at the death, 25% (*n* = 21) were nearby but not present, and 39% (*n* = 32) were not present. Around half of siblings (54%, *n* = 44) reported saying goodbye to their brother/sister before he/she died. Lastly, about half of siblings (54%, *n* = 44) said they would have “done things” differently around the time of the death (e.g., spent more time together, fought less, helped out more). See Table 2 for mother-reported and sibling-reported circumstances surrounding EoL.

Mother and sibling reports on the initial grief interview were concordant regarding whether the sibling said goodbye, χ2 (1, *n* = 76) = 8.29, *p* = 0.004, as well as mother and child report of whether the sibling was present or nearby, χ2 (1, *n* = 79) = 39.38, *p* ≤ 0.001. In contrast, there was poor concordance between mother and sibling reports of whether the sibling anticipated the death, χ2 (1, *n* = 76) = 6.04, *p* = 0.196, with 30% (*n* = 46) of siblings reporting lower rates of anticipation relative to mother-reported sibling anticipation, *t*(75) = 1.52, *p* = 0.018.

### Factors Related to Sibling Internalizing Symptoms, Grief, and Growth

#### Demographic and clinical factors.

There were no significant associations between sibling age and mother-report of sibling internalizing symptoms (*p* = .072) or sibling-reported grief and grief-related growth outcomes (*p* = 0.382 and *p* = .216, respectively). Further, there were no significant associations between months since the child’s death and mother-report of sibling internalizing symptoms (*p* = .297) or sibling-reported grief and grief-related growth outcomes (*p* = 0.534 and *p* = .652, respectively).

#### Mother Report.

Mother-report of sibling internalizing symptoms were significantly above the normative mean, *t*(83) = 4.44, *p* ≤ 0.001 (*M* = 56.01 ± 12.48), with 39% (*n* = 33) in the borderline and/or clinically-significant range. Siblings were reported to have fewer internalizing symptoms if their mother had the opportunity to say goodbye, whereas if the mother had no opportunity (15%), siblings had elevated internalizing symptoms (*M* = 65.5 ± 12.58) in the clinically-significant range, *F*(2, 79) = 4.08, *p* = 0.021. Mother-report of home-based deaths was associated with more sibling growth (*M* = 76.02 ± 16.35) compared to if the sibling died in the hospital (*M* = 68.13 ± 19.04), *t*(80) = 2.02, *p* = 0.047. Finally, based on maternal report, anticipation of the death, sibling presence, who informed the sibling, or if the sibling said goodbye were all unrelated to siblings’ internalizing (*p* = 0.07 – 0.86) and grief outcomes (*p* = 0.23 – 0.73).

#### Sibling Report.

Per sibling-reported circumstances, grief scores were significantly greater for siblings that were present/nearby during the death (*M* = 57.80 ± 19.60) than for those who were not (*M* = 49.95 ± 14.43), *t*(80) = −2.08, *p* = 0.04. Grief-related growth was also significantly greater for siblings that were present/nearby (*M* = 76.00 ± 16.51) than those who were not (*M* = 68.41 ± 16.94), *t*(80) = −2.01, *p* = 0.048. Siblings who said goodbye to their brother/sister had greater grief-related growth (*M* = 76.25 ± 15.60) than those who did not (*M* = 68.38 ± 20.61), *t*(79) = −1.95, *p* = 0.05. Further, grief scores were significantly greater for siblings who wished they had done something differently around the time of the death (*M* = 58.39 ± 19.88) than those who did not (*M* = 49.24 ± 16.59), *t*(80) = −2.24, *p* = 0.028. Similarly, grief-related growth was significantly greater for those who wished they had done things differently (*M* = 76.68 ± 16.02) than those who did not (*M* = 67.55 ± 19.83), *t*(80) = −2.31, *p* = 0.024. Whether the sibling anticipated the death and who informed the sibling were generally unrelated to grief (*p* = 0.23 and *p* = 0.12, respectively) and growth (*p* = 0.44 and *p* = 0.88, respectively).

## Discussion

The experience of grief varies greatly among individuals, especially children and adolescents, and may be affected by circumstances surrounding the death (Lövgren et al., 2018; Nader & Salloum, 2011). Past work on the influence of EoL circumstances, such as anticipating the death and saying goodbye, has primarily focused retrospectively on bereaved adolescents and young adults many years after their brother or sister died from cancer (Lövgren et al., 2018; Rosenberg et al., 2015). In contrast, we focused on more proximal responses after the death, improving the validity of our findings. The fact that several EoL factors were related to internalizing symptoms, grief, and grief-related growth among school-aged and adolescent bereaved siblings shortly after the death may inform improvements to EoL care that may optimize psychosocial outcomes for grieving families.

As expected, mothers reported that bereaved siblings had internalizing symptoms significantly above the normative mean. This is consistent with developmental grief reactions of children and adolescents aged 12 to 18 years (i.e., anxiety, depression, sadness, etc.) (Revet, Laifer, & Raynaud, 2018). About one-third of bereaved siblings had elevated internalizing symptoms irrespective of circumstances at EoL. However, fewer internalizing symptoms were reported for siblings if the parent had the opportunity to say goodbye, whereas siblings whose parent did not say goodbye had scores on average an entire standard deviation above the normative mean in the clinically-significant range. This suggests that saying goodbye may impact parent well-being and possibly how they perceive the psychological adjustment of their surviving children, consistent with theoretical family systems frameworks (Whiteman, McHale, & Soli, 2013). Alternatively, mothers who did not have an opportunity to say goodbye to their child may experience emotional unavailability and prolonged grief, perhaps resulting in a double loss for the surviving sibling given the clinically-significant internalizing symptoms found (Pohlkamp, Kreicbergs, & Sveen, 2019). This may reflect families in which the child’s death came suddenly and was unexpected, leaving less opportunity for communication with the child, or parents who struggled to accept their child would die and had more difficulties overall. Future studies could examine both siblings’ reports of their own internalizing symptoms and parental psychological adjustment to further explore the associations with EoL circumstances surrounding the death.

Interestingly, sibling grief and grief-related growth were generally unrelated to mother-report of circumstances surrounding the death. However, as expected, dying at home was related to sibling growth and thus may be an important predictor of sibling adjustment. This is consistent with work indicating better adjustment among siblings of children who died in the home compared to those who died in the hospital (Mulhern, Lauer, & Hoffmann, 1983). Families who anticipated the death or had more time to prepare may have been more likely to advocate for their child to remain at home, contributing to this finding.

In contrast, sibling reports of the circumstances surrounding the death revealed that siblings who were present/nearby at the time of death, as well as those who expressed regret nearing their sibling’s EoL, had both greater grief and grief-related growth than those who were not present or did not report regrets. Greater grief and growth are not mutually exclusive and can co-occur or have temporal associations with one another (Hogan & De Santis, 1996; Hogan et al., 2014; Hogan & Greenfied, 1991). Indeed, grief itself is not necessarily “bad” and can be adaptive/normative if not traumatic in nature (Revet et al., 2018). This is consistent with a study finding that personal growth followed elevated anxious and depressive symptoms over time among bereaved siblings (Rosenberg et al., 2015). Being present at the death and the potential for situational regret are important factors for clinicians and families to consider at the EoL to enhance positive sibling adjustment. These findings suggest that siblings could be offered a choice to be present when the child dies and be prepared for the experience of the imminent death, providing them with a sense of control at a time when they feel powerless and vulnerable (Nolbris & Hellstrom, 2005; Steele et al., 2013).

Findings further revealed that siblings who had the opportunity to say goodbye to their brother/sister also reported higher grief-related growth than those who did not. This is in keeping with Rosenberg and colleagues work which highlighted that the opportunity to say goodbye was critical to sibling adjustment and psychosocial well-being after the death (Rosenberg et al., 2015). At the same time, our findings suggested that both sibling and mother-report of whether the sibling anticipated the death, based on directly asking, were generally unrelated to their outcomes (Rosenberg et al., 2015). Past work with bereaved siblings, assessed years later as young adults, suggested limited information prior to their sibling’s death was related to unresolved grief (Lövgren, Blyund-Grenklo, Jalmsell, Eilegård Wallin, & Kreicbergs, 2016; Lövgren et al., 2018). Indeed, bereaved siblings report a desire to be informed prior to their brother or sister’s death (Nolbris & Hellstrom, 2005; Steele et al., 2013). Perhaps awareness of the impending death may be more important in predicting persistent or prolonged grief outcomes as opposed to acute responses in the first year. Given the limited literature on sibling adjustment in relation to circumstances surrounding death, further work is needed to address these differences.

Although mother and sibling-reports of EoL circumstances were generally concordant, there was a significant discrepancy between reports of whether siblings anticipated the death, with mothers reporting more siblings were aware of the impending death than siblings acknowledged. This discordance may explain the lack of association between siblings’ anticipation and adjustment, despite past work highlighting its importance. Communication can be challenging at the EoL, with siblings reporting information provided by different sources, such as their parents, healthcare providers, or other family members and friends. Family members often wish to protect each other from distress when a child is seriously ill and limit communication, which has been described as a “conspiracy of silence” (Eaton Russell et al., 2018; Lemus-Riscanevo, Carreño-Moreno, & Arias-Rojas, 2019). Regardless of direct communication from parents or providers, children often have an awareness of the impending death due to information obtained from nonverbal cues, conversations overheard in the hospital or home, and the physical decline of their brother/sister. Our findings support previous recommendations that siblings should be informed in a developmentally appropriate manner (Giovanola, 2005), but, when possible, they also should have the opportunity to participate in events near the end of their brother or sister’s life (Gerhardt et al., 2015). Given these modifiable aspects (e.g., communication about death in developmentally appropriate manner, participate in events near EoL) have often been recommended, they should be implemented systematically by clinical teams when possible.

It is important to note limitations to our study. Despite the relatively larger sample, the smaller number of participating fathers precluded their inclusion in this paper. Future work should specifically recruit and retain fathers to represent the whole family and their unique perspectives. Further, our study focused on siblings who had lost a brother/sister to cancer and may not generalize to other life-limiting illnesses or types of death. Due to conducting research in the home and scheduling delays, a few outlier home visits occurred outside the range of time since bereavement. Given the individual nature of grief, future research should investigate how results compare to more diverse samples of bereaved siblings. Cultural differences and traditions vary in grief and cannot be generalized to all families. As such, providers should assess cultural considerations for each family (Wiener, McConnell, Latella, & Ludi, 2013). In addition, multiple informants and longitudinal follow-up would address limitations from single sources and cross-sectional data. Despite these limitations, this is the first study to examine the impact of EoL circumstances on school-aged children and adolescents within one year after the death of a brother/sister from cancer. Given the frequency of ascertainment bias in past research, recruitment from cancer registries in the first year of the death were strengths within the context of sibling bereavement research.

Overall, findings suggest that the adjustment of bereaved siblings may be affected by certain circumstances surrounding the death of their brother or sister, such as parent and sibling opportunity to say goodbye, presence at the death, and whether or not they wished they had done something differently at the time of death. However, these factors were, for the most part, unique to sibling-report, with few significant predictors of mother-reported sibling experiences. Given this discrepancy, families may benefit from clinician led interventions that facilitate communication amongst family members before and shortly after bereavement (Marsac, Kindler, Weiss, & Ragsdale, 2018). As noted above, the sibling perspective was critical in this study for determining these important findings, yet siblings are often overlooked, both clinically and as research participants. Further work involving siblings is needed to investigate other predictors of adjustment to inform the development of interventions and promote optimal outcomes for bereaved siblings. Results from this study point to certain modifiable aspects of a child’s death that can impact sibling outcomes even a year or more following the death. Because healthcare providers play a pivotal role in providing guidance to families surrounding EoL (Hoffmann et al., 2018; Lövgren, Jalmsell, Eilegård Wallin, Steineck, & Kreicbergs, 2016; Warnick, 2015), it is important to know that what happens at EoL matters for these families during the grief process and for years later. Thus, siblings of children with cancer, may benefit from support and advance planning for their brother’s or sister’s death.

## Figures and Tables

**Table 1.** Family demographic and clinical characteristics (N = 88)

***Demographic Information***	% (*n*)
Sibling Sex	
Male	44.3% (39)
Female	55.7% (49)
Sibling Age (years)	
Mean	12.41
Standard Deviation	2.64
Range	8 – 18
Sibling Race	
White	73% (62)
Black	9% (8)
Asian	7% (6)
Other	11% (10)
Sibling Ethnicity	
Hispanic or Latino	6% (5)
Caregiver Age (years)	
Mean	39.8
Standard Deviation	6.57
Range	27 – 60
Time Since Child’s Death (months)	
Mean	11.57
Standard Deviation	3.48
Range	6 – 24
***Diagnostic Information***	% (*n*)
Diagnostic Category	
Leukemia	23% (20)
Lymphoma	8% (7)
Brain Tumor	28.7% (25)
Solid Tumor	40.2% (35)

**Table 2.** Mother-reported and sibling-reported end-of-life circumstances (N = 88)

	Mother-report % (*n*)	Sibling-report % (*n*)
Anticipated Child’s Death		
Mother	90% (75)	-
Sibling†	78% (65)	57.5% (46)
Days	Median=30 days*	M=44.79, SD=62.99
Who Told Sibling About Child’s Death		
Parent	70% (49)	60% (28)
Medical Staff	10% (7)	2% (1)
Other	20% (14)	38% (18)
Place of Child’s Death		
Home	52% (43)	-
Hospital	43% (36)	-
Other	5% (4)	-
Sibling’s Presence at Death		
Present at Death	35% (29)	36% (30)
Nearby but Not Present	33% (27)	25% (21)
Not Present	33% (27)	39% (32)
Opportunity to Say Goodbye		
Mother	85% (70)	-
Sibling	60% (49)	54% (44)
Would have done things differently		
Yes	-	54% (44)

† Mother and sibling reports of whether the sibling anticipated the death were found to be significantly discordant.

* Median reported due to outliers. Median of 30 days for both mother-reported parent anticipation and mother-reported sibling anticipation.

## Appendix

**Appendix.** Initial Grief Semi-Structured Interview

Question	Response Coded
1. Did you know that your child was going to die before he/she passed away?	Unexpected Expected, but not at that time Expected If expected, how long?
2. Did (the sibling) know?	Unexpected Expected, but not at that time Expected If expected, how long?
3. Who told (the sibling) that his/her brother/sister was going to die?	Parents Deceased child Other family member Friend Medical staff (e.g., MD) More than one of the above
4. Where did your child pass away?	At home In hospital At some other location
5. Was (the sibling) present when his/her brother/sister passed away?	Not present Nearby, but not present Present at death
6. If not present, who told him/her that his/her brother/sister died?	Parents Other family member Friend Medical staff (e.g., MD) More than one of the above
7. Did you have an opportunity to say goodbye to your child before he/she died?	No Yes, spoke to child while alert Yes, spoke to child while unresponsive Yes, sent a letter/picture
